# Le tétanos : une toxi-infection grave encore présente dans le Sud tunisien

**DOI:** 10.48327/mtsi.v6i1.2026.802

**Published:** 2026-01-24

**Authors:** Sabrine BRADAI, Slim BOUAZIZ, Amina HADDED, Najeh BACCOUCH, Mabrouk BAHLOUL, ChokriBEN HAMIDA

**Affiliations:** Service de réanimation polyvalente, CHU Habib Bourguiba Sfax, 3029, Sfax, Tunisie

**Keywords:** Tétanos grave, Réanimation, Complications, Traitement, Vaccin, Mortalité, Tunisie, Afrique du Nord, Severe tetanus, Resuscitation, Complications, Treatment, Vaccine, Mortality, Tunisia, North Africa

## Abstract

**Introduction:**

En dépit d’un recul majeur grâce à la vaccination, le tétanos demeure un problème de santé publique, particulièrement dans les pays en développement. Son épidémiologie en Tunisie n’a pas été actualisée depuis plus de 20 ans. L’objectif de notre travail est d’étudier les caractéristiques épidémiologiques, cliniques, paracliniques, thérapeutiques et évolutives des patients en réanimation atteints du tétanos grave.

**Matériel et méthodes:**

Notre étude était rétrospective et menée dans le service de réanimation polyvalente du CHU Habib Bourguiba de Sfax. Elle s’est étendue sur 19 ans. Nous avons inclus tous les cas de tétanos grave suspectés, selon la définition de l’OMS sur la présence de trismus et/ou de contractures généralisées et/ou de paroxysmes chez un patient non ou mal vacciné.

**Résultats:**

Seize patients ont été inclus. La fréquence était de 0,84 cas/an, l’âge moyen de 61,6 ± 10,1 ans avec une prédominance masculine (sex-ratio à 2,2). La moitié des patients exerçait des professions manuelles, pendant les saisons chaudes, dans les régions rurales. Le symptôme le plus fréquent était une plaie cutanée avec inoculation traumatique (14 patients), au niveau des membres inférieurs (10 patients). Aucun n’était vacciné. Selon la classification d’Ablett, la moitié de nos patients présentait une forme grave ou très grave. Tous nos patients avaient une forme généralisée du tétanos avec un trismus, une raideur de la nuque, une dysphagie et des contractures musculaires généralisées. Treize patients présentaient des paroxysmes, 14 des troubles dysautonomiques dont les plus fréquents étaient les accès de tachycardie (6 patients). La ventilation mécanique a été nécessaire chez 15 patients avec une durée moyenne de 24,9 ± 15,8 jours. La durée totale de séjour a été de 29,7 ± 16,5 jours et 5 patients sont décédés au cours de l’hospitalisation.

**Conclusion:**

Le tétanos demeure une maladie grave avec une lourde morbi-mortalité malgré les avancés thérapeutiques en soins intensifs en Tunisie. La prévention reste la meilleure arme pour l’éradiquer.

## Abstract (en)

**Introduction:**

Although there has been a significant decline in tetanus cases thanks to vaccination, it remains a public health problem, particularly in developing countries. The epidemiology of tetanus in Tunisia has not been updated in over 20 years. Our study aims to examine the epidemiological, clinical, paraclinical, therapeutic, and evolutionary characteristics of patients with severe tetanus in intensive care.

**Materials and methods:**

This retrospective study was conducted in the multi-purpose intensive care unit at Habib Bourguiba University Hospital in Sfax. It spanned 19 years. We included all suspected cases of severe tetanus according to the WHO definition, which includes trismus and/or generalized contractures and/or paroxysms in an unvaccinated or poorly vaccinated patient.

**Results:**

Sixteen patients were included. The frequency was 0.84 cases per year, and the patients’ average age was 61.6 ± 10.1 years. There was a predominance of males, with a sex ratio of 2.2. Half of the patients were manual laborers who worked in rural areas during the hot seasons. The most common symptom was a skin wound with traumatic inoculation in the lower limbs (10 patients), with 14 patients presenting with this symptom. None of the patients had been vaccinated. According to Ablett’s classification, half of our patients had severe or very severe forms of the disease. All patients had generalized tetanus with trismus, neck stiffness, dysphagia, and generalized muscle contractures. Thirteen patients experienced paroxysms, and fourteen had dysautonomic disorders, the most common of which were tachycardia episodes (six patients). Fifteen patients required mechanical ventilation, with an average duration of 24.9 ± 15.8 days. The total length of stay was 29.7 ± 16.5 days, and five patients died during hospitalization.

**Conclusion:**

Despite advances in intensive care in Tunisia, tetanus remains a serious disease with high morbidity and mortality rates. Prevention is the most effective way to eradicate it.

## Introduction

Le tétanos est une maladie infectieuse bactérienne non immunisante due à un bacille à Gram positif anaérobie, *Clostridium tetani* [1,22]. Il s’agit d’une urgence médicale nécessitant une prise en charge en réanimation. Il se manifeste par une rigidité intense et des spasmes musculaires généralisés, pouvant entraîner des complications potentiellement fatales telles que l’insuffisance respiratoire, les troubles dysautonomiques et les infections secondaires [2].

Malgré les progrès réalisés grâce à la vaccination introduite dans les années 1940, le tétanos demeure un problème de santé publique dans de nombreuses régions du monde, notamment dans les pays en développement, où la couverture vaccinale reste insuffisante [15]. Le pronostic des formes graves reste sombre, avec une mortalité élevée, particulièrement dans les zones où l’accès aux soins intensifs est limité [9,16].

En Tunisie, les données épidémiologiques actualisées font défaut depuis plus de 20 ans. Il nous a donc semblé pertinent de mener cette étude afin de décrire les caractéristiques épidémiologiques, cliniques, paracliniques, thérapeutiques et évolutives des patients atteints de tétanos grave admis en réanimation au CHU Habib Bourguiba de Sfax.

## Matériels et méthodes

Notre étude était rétrospective, menée dans le service de réanimation polyvalente du CHU Habib Bourguiba de Sfax, ayant une capacité de 22 lits. La période d’étude s’est étendue sur 19 ans, du 1^er^ janvier 2006 au 31 décembre 2024. Nous avons inclus tous les cas de tétanos grave suspectés selon la définition de l’Organisation mondiale de la santé (OMS) sur la présence de trismus et/ou de contractures généralisées et/ou de paroxysmes chez un patient non ou mal vacciné [14]. Nous avons recueilli les données démographiques, cliniques, paracliniques, thérapeutiques et évolutives au cours du séjour en réanimation. Nous avons calculé le score de gravité d’Ablett [8]. Les données relevées ont été exploitées et analysées par les logiciels EXCEL (Office 2016) et SPSS (version 26). Les variables qualitatives ont été exprimées en fréquence et en pourcentage et les variables quantitatives en moyenne (± écart type et extrêmes).

## Résultats

Notre étude, menée sur une période de 19 ans, a recensé 16 cas de tétanos grave admis en réanimation, soit une incidence moyenne de 0,84 cas par an. L’âge moyen des patients était de 61,6 ± 10,1 ans, avec des extrêmes allant de 46 à 82 ans. La tranche d’âge la plus représentée était celle de 50 à 59 ans (7 patients). Aucun cas de tétanos néonatal n’a été enregistré. Une prédominance masculine a été observée, avec un sex-ratio de 2,2. Dix patients ne présentaient pas d’antécédents médicaux connus, et aucun n’était vacciné. Tous étaient originaires de zones rurales du Sud tunisien. Une activité professionnelle manuelle exposant à un risque de traumatisme, en particulier durant les saisons chaudes, était retrouvée chez la moitié des patients. Les données relatives aux professions, aux portes d’entrée infectieuse, aux agents traumatiques et à la topographie des lésions sont synthétisées dans le Tableau I.

**Tableau I:** Données épidémiologiques et cliniques des patients atteints de tétanos grave à l’admission en réanimation

Données	Effectif	Fréquence (%)
Porte d’entrée infectieuse		
cutanée	14	87,5
inconnue	2	12,5
Agent causal		
clou	9	56,3
barreau métallique	1	6,3
épine de palmier	1	6,3
épine de poisson	1	6,3
griffures de poule	1	6,3
morceau de bois	1	6,3
non identifié	2	12,2
Siège		
membres inférieurs	10	62,5
membres supérieurs	3	18,8
céphalique	1	6,3
non identifié	2	12,4
Profession		
maçon	3	18,8
menuisier	1	6,3
pêcheur	2	12,6
topographe	1	6,3
agriculteur	1	6,3
femme au foyer	5	31,3
inconnue	3	18,8
Signes cliniques		
trismus	16	100
contractures généralisées	16	100
paroxysmes	13	81,3
faciès sardonique	16	100
dysphagie	16	100
raideur de la nuque	16	100
hyperlordose lombaire	3	18,8
dysautonomie	14	87,5
tachycardie	6	37,5
bradycardie	1	6,25
hypertension	4	25
hypersudation	4	25
hyperthermie	2	12,5
hypersialorrhée	4	25

La durée d’incubation moyenne était de 9,6 ± 5,2 jours avec des extrêmes allant de 3 à 20 jours. Quatre patients avaient une durée d’incubation inférieure à 7 jours. La durée d’invasion était de 2,4 ± 1,3 jours avec des extrêmes allant de 1 à 5 jours. Pour 4 patients, cette durée était inférieure à 2 jours.

Tous nos patients présentaient une forme généralisée du tétanos avec un trismus, une raideur de la nuque, une dysphagie et des contractures musculaires généralisées. Treize patients montraient des paroxysmes et 14 des troubles dysautonomiques, dont les plus fréquents étaient les accès de tachycardie (6 patients) (Tableau I). Selon la classification d’Ablett [8], la moitié de nos patients présentait une forme grave ou très grave (Tableau II).

**Tableau II:** Répartition des patients selon la classification d’Ablett à l’admission [8]

Classification d’Ablett	Caractéristiques cliniques	Nombre de cas
Atteinte légère	Trismus léger à modéré; spasticité générale; aucune gêne respiratoire; aucun spasme; dysphagie légère ou inexistante	1
Atteinte modérée	Trismus modéré; rigidité bien marquée; spasmes légers à modérés mais courts; gêne respiratoire modérée avec augmentation de la fréquence respiratoire > 30/min; dysphagie légère	7
Atteinte grave	Trismus grave; spasmes réflexes prolongés; fréquence respiratoire > 40 cycles/min; crises d’apnée; dysphagie grave; tachycardie > 120/min]	3
Atteinte très grave	Troubles autonomiques violents de grade III touchant le système cardiovasculaire; hypertension grave et tachycardie alternant avec une hypotension relative et une bradycardie, chacune pouvant être persistante	5

Concernant la prise en charge thérapeutique, tous les patients ont bénéficié d’un conditionnement initial comprenant un cathétérisme veineux central et artériel, ainsi qu’un sondage gastrique et vésical. Au cours du séjour en réanimation, 14 patients ont reçu une nutrition artificielle par voie entérale. Un traitement préventif des complications fréquentes en réanimation a été instauré systématiquement, incluant la prévention de l’ulcère de stress, des maladies thromboemboliques et des lésions cutanées trophiques. Quinze patients ont nécessité une ventilation mécanique, d’une durée moyenne de 24,9 ± 15,8 jours (extrêmes : 1 à 57 jours). Une trachéotomie a été réalisée chez 13 patients, en raison d’un trismus, dans un délai moyen post-hospitalisation de 1,7 ± 1,6 jours (extrêmes : 0 à 6 jours) après l’admission.

Le deuxième volet thérapeutique a consisté en l’identification, la désinfection et/ou le débridement de la porte d’entrée infectieuse. Aucun patient n’a nécessité de geste chirurgical majeur. Concernant l’antibiothérapie ciblant *C. tetani*, tous les patients ont reçu du métronidazole, seul ou en association avec de la pénicilline G ou du cefotaxime, pour une durée moyenne de 9,5 ± 4,8 jours.

Tous les patients ont également bénéficié d’une immunisation antitétanique combinant une vaccination par anatoxine tétanique et une sérothérapie (SAT), avec une dose moyenne de 2 570 UI administrée par voie intramusculaire (extrêmes : 1 500 à 5 000 UI). La sérothérapie a été réalisée à l’aide de Tetanus Gamma^®^ 250 UI, une immunoglobuline humaine antitétanique spécifique, disponible en Tunisie sous forme de seringue préremplie de 1 ml.

Pour le contrôle des spasmes musculaires, le diazépam (Valium^®^) a été prescrit à tous les patients, pendant une durée moyenne de 12,4 ± 7 jours. Le baclofène par voie orale a été utilisé chez la moitié des patients, pour une durée moyenne de 15,4 ± 8,9 jours. En cas de contractures persistantes, le phénobarbital (Gardénal^®^) a été administré chez 3 patients. Une sédation intraveineuse a été instaurée chez 14 patients, associant le midazolam (Hypnovel^®^) et un morphinique (fentanyl), pourune durée moyenne de 15,1 ± 9,9 jours (extrêmes : 1 à 31 jours). Une curarisation a été nécessaire chez 8 patients. Chez les patients présentant des troubles dysautonomiques, certains médicaments ont été prescrits tels que le sulfate de magnésium (5 cas), la nicardipine (2 cas) et l’amiodarone (1 cas). L’évolution a été marquée par une morbidité importante, avec une durée moyenne de séjour en réanimation de 29,7 ± 16,5 jours. Les principales complications observées étaient les infections associées aux soins (12 patients) (Tableau III). Malgré la prise en charge multidisciplinaire, l’évolution a été associée à une mortalité élevée (5 patients), dont la cause la plus fréquente a été les troubles dysautonomiques cardiaques (2 patients).

**Tableau III:** Les complications présentées par les patients atteints de tétanos grave au cours du séjour en réanimation

Complications	Effectif	Fréquence (%)
**Complications infectieuses**	**12**	**75**
pulmonaire	10	62,5
urinaire	7	43,8
endovasculaire	4	25
**Complications dysautonomiques**	**14**	**87,5**
fibrillation ventriculaire	1	6,3
cardiomyopathie de Takotsubo	1	6,3
tachycardie	6	37,5
bradycardie	1	6,3
pic hypertensif	2	12,5
fièvre non septique	3	18,8
**Complications respiratoires**	**10**	**62,5**
atélectasie	6	37,5
abcès pulmonaire	1	6,3
épanchement pleural	2	12,5
pneumothorax	1	6,3
**Complications digestives**	**4**	**25**
hématémèse	2	12,5
cholécystite aiguë	1	6,3
diarrhée	4	25
constipation	2	12,5
**Complications ostéoarticulaires**	**5**	**31,3**
dénutrition	5	31,3
neuromyopathie de réanimation	5	31,3
ostéomes	5	31,3
escarres	2	12,5
rétraction musculo-tendineuse	4	25
**Insuffisance rénale aiguë**	**5**	**31,3**
**Maladies thromboemboliques**	**2**	**12,5**
thrombose veineuse profonde	1	6,3
embolie pulmonaire	2	12,5

## Discussion

À notre connaissance, cette étude constitue la première série tunisienne décrivant les formes graves de tétanos provenant du sud du pays et admises en réanimation, offrant ainsi une actualisation du profil épidémiologique de cette maladie sur les deux dernières décennies.

Nos résultats montrent que l’incidence du tétanos en Tunisie a considérablement diminué, passant de 35 cas annuels dans les années 1980 à 3 cas en 2015. Cette diminution est largement attribuable au renforcement progressif des stratégies vaccinales mises en place par le programme national de vaccination.

La vaccination infantile systématique, introduite en 1978 en Tunisie est structurée autour d’un schéma primaire à deux, trois et six mois, suivi de rappels à 18 mois, 6 ans et 12 ans. Elle constitue la mesure la plus déterminante dans le contrôle durable du tétanos. Cette stratégie a été consolidée par l’instauration, à partir de 1984, de la vaccination systématique des femmes enceintes, l’élargissement en 1988 de la couverture vaccinale à l’ensemble des femmes en âge de procréer, puis l’adoption en 1992 de la politique de protection à la naissance, qui a permis l’élimination quasi complète du tétanos néonatal [13]. En Tunisie, le calendrier vaccinal actualisé en 2025 recommande également des rappels à 18 ans puis tous les 10 ans à l’âge adulte, mesure indispensable pour maintenir une immunité protectrice, en particulier chez les sujets âgés dont la réponse vaccinale tend à décliner avec le temps. Il est également important de rappeler que le service de réanimation étudié, dédié exclusivement aux patients adultes, ne prend pas en charge les nouveau-nés, les formes néonatales étant par ailleurs considérées comme éliminées en Tunisie depuis les années 1990.

Malgré ces acquis, le tétanos persiste sous forme de cas sporadiques, principalement chez les adultes insuffisamment vaccinés ou n’ayant pas bénéficié des rappels décennaux. Cela est illustré dans notre série, dans laquelle les patients les plus touchés étaient des hommes âgés de 50 à 60 ans, non vaccinés. Ce profil reflète la persistance de lacunes vaccinales chez les générations masculines plus âgées, qui n’ont pas bénéficié des campagnes de rattrapage ciblant les femmes en âge de procréer, ni de la vaccination systématique des hommes soumis au service militaire.

Notre étude montre également que la prévalence est plus élevée en milieu rural, en lien avec un accès limité aux soins et une application insuffisante des mesures prophylactiques [21]. Par ailleurs, les travailleurs agricoles sont particulièrement exposés, en raison de leur contact fréquent avec les réservoirs de spores tétaniques présents dans les sols riches en matière organique des zones chaudes et tempérées [10]. Cette exposition, associée à une activité physique intense en période estivale, explique la vulnérabilité accrue des professions manuelles, avec une porte d’entrée infectieuse souvent localisée aux membres inférieurs.

Sur le plan clinique, tous nos patients présentaient à l’admission un tétanos généralisé grave avec un trismus, une raideur de la nuque, une dysphagie et des contractures musculaires généralisées. Au cours du séjour en réanimation, la plupart des patients avaient des paroxysmes et des troubles dysautonomiques dont les plus fréquents étaient les accès de tachycardie. Différents scores pronostiques ont été établis afin d’évaluer la gravité du tétanos [8,17,23]. Le score d’Ablett est le plus couramment utilisé. Dans notre étude, la moitié de nos patients présentait une forme grave ou très grave. Nous n’avons pas fait ressortir les facteurs de mauvais pronostic, étant donné la taille réduite de notre échantillon. Dans la littérature, les facteurs de mauvais pronostic sont : les âges extrêmes, une période d’incubation inférieure à 7 jours, une période d’invasion inférieure à 2 jours, la présence à l’admission de fièvre, de tachycardie, de spasmes et certaines portes d’entrée infectieuse (puerpérale, intraveineuse, postopératoire, brûlures) [3,21].

La prise en charge des patients de notre étude a suivi les principes standards de réanimation des formes graves de tétanos, combinant un soutien ventilatoire prolongé, la trachéotomie lorsque l’intubation prolongée était nécessaire [4] et le monitorage cardio-respiratoire intensif. Ces mesures sont essentielles pour prévenir la dépression respiratoire, principale cause de mortalité dans cette pathologie [20]. La nutrition entérale précoce, associée à la prévention systématique des complications trophiques, thromboemboliques et des ulcères de stress, a permis de maintenir l’intégrité immunitaire et de limiter le risque d’infections nosocomiales.

Le traitement étiologique a consisté en la désinfection et le débridement de la porte d’entrée infectieuse, associés à une antibiothérapie ciblée contre *C. tetani*, conformément aux recommandations de l’OMS, et à une immunisation antitétanique combinant anatoxine et sérothérapie [14].

La sérothérapie a pour but de neutraliser la toxine circulante et la toxine libre au niveau de la plaie. En effet, la toxine déjà fixée sur les neurones n’est pas neutralisée par ce traitement. Les immunoglobulines antitétaniques spécifiques d’origine humaine représentent la sérothérapie idéale. L’OMS et les Centres pour le contrôle et la prévention des maladies des États-Unis d’Amérique recommandent son administration à la dose de 500 à 1 000 UI par voie intramusculaire [7,14]. Dans notre série, l’immunothérapie a été réalisée exclusivement par voie intramusculaire avec une dose moyenne de 2 570 UI. L’administration intrathécale d’antitoxine dans le traitement du tétanos reste controversée. Plusieurs études [5,11,12] ont suggéré une supériorité de ce traitement par rapport à l’administration intramusculaire en termes de réduction de la mortalité ou de la durée d’hospitalisation. D’autres travaux, notamment des essais randomisés [24], n’ont pas retrouvé de bénéfice significatif. Par ailleurs, des préoccupations liées aux effets indésirables potentiels, comme la paraplégie réversible observée à fortes doses [19], ainsi que la variabilité des protocoles utilisés, limitent la généralisation de cette approche. Ainsi, malgré des résultats prometteurs, la voie intrathécale n’est pas systématiquement recommandée et son indication devrait être évaluée au cas par cas, en tenant compte des ressources disponibles et du profil du patient.

Le tétanos n’étant pas une maladie immunisante, il faut débuter la vaccination. Celle-ci est réalisée par une injection d’anatoxine en un site différent de l’injection du SAT. Elle sera répétée ultérieurement deux fois à un mois d’intervalle. Afin de contrôler les spasmes, les benzodiazépines sont les médicaments de choix, utilisés en 1^ère^ intention dans la sédation du fait de leur propriété myorelaxante en rapport avec un effet agoniste du GABA [18]. Le diazépam est la molécule la plus utilisée. Sa posologie est de 3 à 5 mg/kg/jour en perfusion continue avec des bolus de 5 à 10 mg en cas de paroxysmes. Le midazolam, à la fois sédatif, anxiolytique, myorelaxant et anticonvulsivant, peut être utilisé en perfusion continue à la dose de 5-15 mg/h en raison de sa demi-vie courte. Les barbituriques (phénobarbital) atténuent les contractures avec des posologies élevées (1-2 mg/ kg/j en IV). Les autres myorelaxants recommandés sont le baclofène, un agoniste de synthèse des récepteurs GABA de type B médullaires administré par voie orale ou intrathécale, et le dantrolène, un myorelaxant spécifique du muscle strié squelettique. Les curares sont indiqués de façon ponctuelle ou prolongée lorsque les paroxysmes ne sont pas contrôlés par les sédatifs [4].

Afin de contrôler le syndrome dysautonomique, nous avons eu recours au sulfate de magnésium pour ses propriétés vasodilatatrices et sa capacité à réduire la libération de catécholamines [6].

La toxine restant fixée pendant 3 à 4 semaines, la résolution progressive des contractures ainsi que la reprise de la déglutition et de l’alimentation normale entraînent une prolongation significative du séjour hospitalier. Cela explique la morbidité importante et les complications fréquentes chez nos patients, dominées par les infections nosoco-miales surtout broncho-pulmonaires. Elles sont favorisées par les fausses routes, la ventilation mécanique prolongée et l’usage des myorelaxants [8]. De plus, les complications cardiovasculaires résultent de la dysautonomie neurovégétative, la survenue de l’ischémie myocardique et les accidents thromboemboliques. Les complications trophiques et ostéoarticulaires telles que rétractions musculo-tendineuses, ostéomes, fractures et escarres, sont aussi fréquentes et favorisées par le séjour long, l’absence de kinésithérapie et les spasmes.

Malgré la prise en charge multidisciplinaire, le taux de létalité a été de 31 % (5/16) dans notre étude. Certes, il est en diminution grâce à l’amélioration des soins intensifs (il était de 63 % dans les années 80). Mais il reste encore élevé par rapport aux pays développés (entre 10 % et 30 %) [25], ce qui s’explique par la gravité des patients dès l’admission.

Ainsi, la prévention demeure la meilleure arme contre le tétanos, reposant sur la vaccination dès l’enfance avec rappels décennaux à l’âge adulte, les campagnes de rattrapage ciblant les hommes à risque, l’asepsie rigoureuse des plaies et des soins obstétricaux, ainsi que l’administration précoce d’immunoglobulines spécifiques en cas de plaies souillées.

## Conclusion

Le tétanos demeure une maladie grave avec une lourde morbi-mortalité malgré les avancées thérapeutiques en soins intensifs en Tunisie, en Afrique du Nord et dans les zones tropicales. La prévention reste la principale stratégie de contrôle de cette maladie, avec pour objectif l’élimination des formes néonatales et la réduction des cas graves chez l’adulte.

## Financement

## Remerciements

Nous tenons à exprimer notre profonde gratitude au Professeur Mounir Bouaziz pour sa contribution à la rédaction et la révision de cet article.

## Contribution des auteurs et autrices

Sabrine Bradai : conception, rédaction, révision et validation du manuscrit. Slim Bouaziz : rédaction et révision du manuscrit. Amina Hadded : rédaction et révision du manuscrit. Najeh Baccouch : révision et validation du manuscrit. Mabrouk Bahloul : rédaction, révision et validation du manuscrit. Chokri Ben Hamida : révision et validation du manuscrit.

## Déclaration de liens d’intérêts

Aucun lien d’intérêt n’a été déclaré.
