# Supplementary material for: Tetanus: a serious toxic infection still present in southern Tunisia
Source: Med Trop Sante Int. 2026 Jan 24;6(1):mtsi.v6i1.2026.802. [Article in French] doi: 10.48327/mtsi.v6i1.2026.802 (PMC13216918; doi:10.48327/mtsi.v6i1.2026.802)
Supplement: Related texts

# Research article: Tetanus: a serious toxic infection still present in southern Tunisia

## Introduction

Tetanus is a bacterial infectious disease that does not confer immunity. It is caused by the Gram-positive, anaerobic bacillus *Clostridium tetani* [1, 22]. It is a medical emergency requiring intensive care. Symptoms include intense muscle rigidity and spasms, which can lead to respiratory failure, dysautonomic disorders, and secondary infections [2].

Despite progress thanks to the introduction of vaccines in the 1940s, tetanus remains a public health concern in many parts of the world, particularly in developing countries where vaccination coverage is insufficient [15]. The prognosis for severe cases is poor, with high lethality rates, especially in areas with limited access to intensive care [9,16].

In Tunisia, there has been a lack of updated epidemiological data for over 20 years. Therefore, we felt it was appropriate to conduct this study in order to describe the epidemiological, clinical, paraclinical, therapeutic and evolutionary characteristics of patients with severe tetanus admitted to the intensive care unit at Habib Bourguiba University Hospital in Sfax.

## Materials and Methods

Our retrospective study was conducted in the multipurpose intensive care unit (ICU) of the Habib Bourguiba University Hospital in Sfax, which has a capacity of 22 beds. The study spanned 19 years, from January 1, 2006, to December 31, 2024. We included all cases suspected to be severe tetanus, according to the World Health Organization (WHO) definition, which includes trismus and/ or generalized contractures and/or paroxysms in an unvaccinated or poorly vaccinated patient [14]. We collected demographic, clinical, paraclinical, therapeutic, and evolutionary data during the intensive care unit stay. We calculated the Ablett severity score [8]. We processed and analyzed the collected data using Excel (Office 2016) and SPSS (version 26) software. Qualitative variables were expressed as frequencies and percentages, and quantitative variables were expressed as means ± standard deviation and extremes.

## Results

Our 19-year study identified 16 cases of severe tetanus admitted to the intensive care unit, representing an average incidence of 0.84 cases per year. The average patient age was 61.6 ± 10.1 years, ranging from 46 to 82 years. The most represented age group was 50 to 59 years old (seven patients). No cases of neonatal tetanus were recorded. A male predominance was observed, with a sex ratio of 2.2. Ten patients had no known medical history, and none had received the tetanus vaccine. All patients were from rural areas in southern Tunisia. Half of the patients had manual occupations that exposed them to a risk of trauma, particularly during the hot seasons. Data on occupations, routes of infection, traumatic agents, and lesion topography are summarized in Table I.

**Table I:** Epidemiological and clinical data for patients with severe tetanus upon admission to the intensive care unit

Data	Headcount	Frequency (%)
Route of infection		
skin	14	87,5
inconnue	2	12,5
Causative agent		
nail	9	56,3
metal bar	1	6,3
palm tree thorn	1	6,3
fish bone	1	6,3
chicken scratch	1	6,3
piece of wood	1	6,3
unidentified	2	12,2
Route of infection		
lower limbs	10	62,5
upper limbs	3	18,8
cephalic	1	6,3
unidentified	2	12,4
Occupation		
bricklayer	3	18,8
carpenter	1	6,3
fisherman	2	12,6
surveyor	1	6,3
farmer	1	6,3
housewife	5	31,3
unknown	3	18,8
Clinical signs		
trismus	16	100
generalized contractures	16	100
paroxysms	13	81,3
sardonic facies	16	100
dysphagia	16	100
stiff neck	16	100
lumbar hyperlordosis	3	18,8
dysautonomia	14	87,5
tachycardia	6	37,5
bradycardia	1	6,25
hypertension	4	25
hypersudation	4	25
hyperthermia	2	12,5
hypersalivation	4	25

The average incubation period was 9.6 days, ranging from three to 20 days. Four patients had an incubation period of less than seven days. The invasion duration was 2.4 ± 1.3 days, ranging from one to five days. For four patients, this duration was less than two days.

All of our patients had generalized tetanus with trismus, neck stiffness, dysphagia, and generalized muscle contractures. Thirteen patients experienced paroxysms, and 14 patients had dysautonomic disorders, the most common of which were tachycardia episodes (six patients) (Table I). According to Ablett’s classification [8], half of our patients had a severe or very severe form (Table II).

**Table II:** Distribution of patients according to Ablett’s classification on admission [8]

Ablett classification	Clinical characteristics	Number of cases
Mild impairment	Mild to moderate trismus; general spasticity; no respiratory distress; no spasms; mild or no dysphagia	1
Moderate impairment	Moderate trismus; marked rigidity; mild to moderate but short spasms; moderate respiratory distress with increased respiratory rate > 30/min; mild dysphagia	7
Severe involvement	Severe trismus; prolonged reflex spasms; respiratory rate > 40 cycles/ min; apnea attacks; severe dysphagia; tachycardia > 120/min]	3
Very severe involvement	Severe grade III autonomic disorders affecting the cardiovascular system; severe hypertension and tachycardia alternating with relative hypotension and bradycardia, each of which may be persistent	5

All patients underwent initial conditioning, including central and arterial venous catheterization, as well as gastric and bladder catheterization. Fourteen patients received artificial enteral nutrition during their stay in intensive care. We systematically instituted preventive treatment for common intensive care complications, including stress ulcer prevention, thromboembolic disease prevention, and trophic skin lesion prevention. Fifteen patients required mechanical ventilation for an average timeframe of 24.9 ± 15.8 days (range 1–57 days). Thirteen patients underwent tracheostomy due to trismus within an average post-hospitalization timeframe of 1.7 ± 1.6 days (range: 0 to 6 days).

The second part of the treatment consisted of identifying, disinfecting, and/or debriding the site of infection. No patient required major surgery. Regarding antibiotic therapy targeting *C. tetani*, all patients received metronidazole, either alone or in combination with penicillin G or cefotaxime, for an average of 9.5 ± 4.8 days.

All patients received tetanus immunization through a combination of tetanus toxoid vaccination and serotherapy (SAT). An average dose of 2,570 IU was administered intramuscularly, ranging from 1,500 to 5,000 IU. Serotherapy was performed using Tetanus Gamma® 250 IU, a specific human tetanus immunoglobulin available in Tunisia as a pre-filled 1-ml syringe.

To control muscle spasms, diazepam (Valium®) was prescribed to all patients for an average of 12.4 ± 7 days. Half of the patients received oral baclofen for an average of 15.4 ± 8.9 days. Three patients received phenobarbital (Gardenal®) for persistent contractures. Fourteen patients received intravenous sedation combining midazolam (Hypnovel®) and a morphine derivative (fentanyl) for an average of 15.1 ± 9.9 days (range: 1–31 days). Curarization was necessary for eight patients. Certain drugs were prescribed to patients with dysautonomic disorders, including magnesium sulfate (five cases), nicardipine (two cases), and amiodarone (one case).

The disease course was marked by significant morbidity, with an average intensive care stay of 29.7 ± 16.5 days. The main complications observed were healthcare-associated infections in 12 patients (Table III). Despite multidisciplinary management, the outcome was associated with high mortality (five patients); the most common cause was cardiac dysautonomia (two patients).

**Table III:** Complications experienced by patients with severe tetanus during their stay in intensive care

Complications	Headcount	Frequency (%)
**Infectious complications**	**12**	**75**
pulmonary	10	62,5
urinary	7	43,8
endovascular	4	25
**Dysautonomic complications**	**14**	**87,5**
ventricular fibrillation	1	6,3
Takotsubo cardiomyopathy	1	6,3
tachycardia	6	37,5
bradycardia	1	6,3
hypertensive peak	2	12,5
non-septic fever	3	18,8
**respiratory complications**	**10**	**62,5**
atelectasis	6	37,5
lung abscess	1	6,3
pleural effusion	2	12,5
pneumothorax	1	6,3
**Digestive complications**	**4**	**25**
hematemesis	2	12,5
acute cholecystitis	1	6,3
diarrhea	4	25
constipation	2	12,5
**Osteoarticular complications**	**5**	**31,3**
malnutrition	5	31,3
resuscitation neuromyopathy	5	31,3
osteomas	5	31,3
pressure sores	2	12,5
muscle and tendon retraction	4	25
**Acute renal failure**	**5**	**31,3**
**Thromboembolic diseases**	**2**	**12,5**
deep vein thrombosis	1	6,3
pulmonary embolism	2	12,5

## Discussion

To our knowledge, this study is the first Tunisian series to describe severe cases of tetanus originating in southern Tunisia and requiring intensive care. This study provides an update on the epidemiological profile of this disease over the last two decades.

Our results show that the incidence of tetanus in Tunisia decreased significantly from 35 cases per year in the 1980s to three cases in 2015. This decrease is largely due to the gradual improvement of vaccination strategies implemented by the national immunization program.

Tunisia introduced routine childhood vaccination in 1978. It is structured around a primary schedule at two, three, and six months, followed by boosters at 18 months, six years, and 12 years. This is the most decisive measure in the sustainable control of tetanus. This strategy was further strengthened by the 1984 introduction of routine vaccination of pregnant women, the 1988 extension of vaccination coverage to all women of childbearing age, and the 1992 adoption of the birth protection policy, which nearly eliminated neonatal tetanus [13]. Tunisia’s updated 2025 vaccination schedule also recommends boosters at age 18 and then every 10 years in adulthood. This is an essential measure for maintaining protective immunity, particularly in older individuals, whose vaccine response tends to decline over time. It is important to note that the intensive care unit studied exclusively treats adult patients and does not treat newborns because neonatal forms of the disease have been considered eliminated in Tunisia since the 1990s.

Despite these achievements, tetanus persists in sporadic cases, primarily affecting adults who are inadequately vaccinated or have not received ten-yearly booster shots. Our series illustrates this, as the most affected patients were unvaccinated men aged 50 to 60. This reflects the persistence of vaccination gaps among older generations of men who did not benefit from catch-up campaigns targeting women of childbearing age or the systematic vaccination of men undergoing military service.

Our study also shows that the prevalence of tetanus is higher in rural areas due to limited access to healthcare and the insufficient application of prophylactic measures [21]. Furthermore, agricultural workers are particularly vulnerable due to their frequent contact with soil rich in organic matter, which contains tetanus spores, in warm and temperate zones [10]. This exposure, combined with intense physical activity during the summer months, explains why manual laborers are more vulnerable, with the infection often entering the body through the lower limbs.

All of our patients presented with severe generalized tetanus, trismus, neck stiffness, dysphagia and generalized muscle contractures on admission. During their stay in intensive care, most patients experienced paroxysms and dysautonomic disorders, the most common of which were episodes of tachycardia. Various prognostic scores have been developed to evaluate the severity of tetanus [8,17,23]. The Ablett score is the most commonly used. In our study, half of our patients had a severe or very severe form of the disease. Given the small size of our sample, we did not identify any factors associated with a poor prognosis. The literature identifies several factors associated with poor prognosis, including extreme ages, an incubation period of less than seven days, an invasion period of less than two days, and the presence of fever, tachycardia and spasms at admission, as well as certain routes of infection (puerperal, intravenous, postoperative and burns) [3,21].

Patients in our study were managed according to the standard principles of resuscitation for severe tetanus, which involves providing prolonged ventilatory support and performing tracheostomy when prolonged intubation is necessary [4], as well as intensive cardiorespiratory monitoring. These measures are essential to prevent respiratory depression, which is the leading cause of mortality in this disease [20]. The combination of early enteral nutrition and systematic prevention of trophic and thromboembolic complications, as well as stress ulcers, helped to maintain immune integrity and limit the risk of nosocomial infections.

The etiological treatment consisted of disinfecting and debriding the infected site, combined with targeted antibiotic therapy against *C. tetani* in accordance with WHO recommendations, as well as tetanus immunization involving a combination of toxoid and serum therapy [14].

The aim of serotherapy is to neutralize the circulating and free toxins in the wound. However, the toxin already attached to the neurons is not neutralized by this treatment. Specific human tetanus immunoglobulins are the ideal serotherapy. The WHO and the US Centers for Disease Control and Prevention recommend an intramuscular dose of 500–1,000 IU [7,14]. In our series, immunotherapy was administered exclusively by the intramuscular route at an average dose of 2,570 IU. The intrathecal administration of antitoxin in the treatment of tetanus remains controversial. Several studies [5,11,12] have suggested that this treatment reduces mortality or length of hospital stay more effectively than intramuscular administration. However, other studies, including randomized trials, have not found any significant benefit [24]. Furthermore, concerns about potential adverse effects, such as reversible paraplegia observed at high doses [19], as well as variability in the protocols used, limit the widespread use of this approach. Thus, despite promising results, the intrathecal route is not routinely recommended, and its use should be evaluated on a case-by-case basis, taking into account available resources and the patient’s profile.

As tetanus does not confer immunity, vaccination must be initiated. This involves injecting the toxoid into a site different from the SAT injection site. This will be repeated twice at Sone-month intervals.

Benzodiazepines are the drugs of choice for controlling spasms and are used as the primary sedative due to their muscle relaxant properties, which are related to their GABA agonist effect [18]. Diazepam is the most commonly used benzodiazepine. The recommended dosage is 3–5 mg/kg/day via continuous infusion, with boluses of 5–10 mg administered during paroxysms. Midazolam, which has sedative, anxiolytic, muscle relaxant and anticonvulsant properties, can be administered via continuous infusion at a dose of 5-15 mg/h due to its short half-life. Barbiturates (e.g. phenobarbital) can reduce contractures at high doses (1-2 mg/kg/day intravenously). Other recommended muscle relaxants include baclofen, which is a synthetic agonist of medullary GABA type B receptors and is administered either orally or intrathecally, and dantrolene, which is a muscle relaxant that acts specifically on striated skeletal muscle. Curare agents are indicated on an ad hoc or prolonged basis when paroxysms are not controlled by sedatives [4].

To control dysautonomia syndrome, magnesium sulfate is used for its vasodilatory properties and ability to reduce catecholamine release [6].

As the toxin remains fixed for three to four weeks, the gradual resolution of contractures and resumption of swallowing and normal feeding lead to a significant prolongation of hospital stays. This explains the high morbidity and frequent complications in our patients, which are dominated by nosocomial infections, especially bronchopulmonary infections. These are exacerbated by aspiration, prolonged mechanical ventilation and the use of muscle relaxants [8]. In addition, cardiovascular complications result from neurovegetative dysautonomia, the onset of myocardial ischemia and thromboembolic events. Trophic and osteoarticular complications, such as muscle and tendon contractures, osteomas, fractures and pressure sores, are also common, and are exacerbated by prolonged hospital stays, a lack of physical therapy and spasms.

Despite multidisciplinary care, the case fatality rate was 31% (5/16) in our study. Admittedly, this figure is decreasing thanks to improvements in intensive care; it stood at 63% in the 1980s. However, it remains high compared to developed countries, where it is between 10% and 30% [25]. This difference can be explained by the severity of patients’ conditions upon admission.

Prevention therefore remains the most effective way to combat tetanus, through childhood vaccination and booster shots every ten years in adulthood, as well as catch-up campaigns targeting at-risk men, rigorous aseptic treatment of wounds and obstetric care, and the early administration of specific immunoglobulins in cases of contaminated wounds.

## Conclusion

Tetanus remains a serious disease with high morbidity and case fatality rates, even in Tunisia, North Africa and other tropical regions where there have been advances in intensive care. The main strategy for controlling this disease is prevention, with the goal of eliminating neonatal forms and reducing severe cases in adults.

## Funding

The authors declare no funding related to this work.

## Acknowledgements

We would like to express our deep gratitude to Professor Mounir Bouaziz for his contribution to writing and revising this article.

## Authors’ contribution

Sabrine Bradai: design, writing, revision and validation of the manuscript. Slim Bouaziz: writing and revision of the manuscript. Amina Hadded: writing and revision of the manuscript. Najeh Baccouch: revision and validation of the manuscript. Mabrouk Bahloul: writing, revision and validation of the manuscript. Chokri Ben Hamida: revision and validation of the manuscript.

## Conflicts of interest

The authors declare no conflicts of interest.
